# Absolute quantification of VBNC state formation and resuscitation in alcohol-producing *Klebsiella pneumoniae*

**DOI:** 10.3389/fcimb.2025.1627943

**Published:** 2025-10-06

**Authors:** Shuo Zhao, Chenpu Dou, Yang Yang, Jiazheng Wang, Meng Li, Bin Yang, Jing Yuan, Jian Zhang

**Affiliations:** ^1^ Department of Bacteriology, Capital Center for Children’s Health, Capital Medical University, Capital Institute of Pediatrics, Beijing, China; ^2^ Department of Neurosurgery, Capital Center for Children’s Health, Capital Medical University, Capital Institute of Pediatrics, Beijing, China; ^3^ Department of Clinical Laboratory Medicine, The First Affiliated Hospital of Shandong First Medical University & Shandong Provincial Qianfoshan Hospital, Shandong Medicine and Health Key Laboratory of Laboratory Medicine, Jinan, Shandong, China; ^4^ Department of Neurosurgery, North China University of Science and Technology Affiliated Hospital, Tangshan, China; ^5^ Department of Neurology, North China University of Science and Technology Affiliated Hospital, Tangshan, China

**Keywords:** HiAlc *Kpn*, VBNC state, ddPCR, viable cell counting, resuscitation, ciprofloxacin

## Abstract

**Introduction:**

Many bacterial species in the viable but non-culturable (VBNC) state pose a considerable risk to public health, primarily because of their ability to persist and resuscitate in the environment and their enhanced ability to escape detection. Our research revealed that high alcohol-producing *Klebsiella pneumoniae* (HiAlc *Kpn*) could cause non-alcoholic fatty liver disease. Notably, HiAlc *Kpn* is prevalent in the gut microbiome, and tracing pathogens in intestinal samples requires identifying and quantifying viable cells.

**Methods:**

For precise quantification, we optimized PMA concentration between 5 μM and 200 μM and adjusted the incubation time from 5 to 30 minutes. Subsequently, we established real-time quantitative PCR (qPCR) and droplet digital PCR (ddPCR) methods to count HiAlc *Kpn* viable cells.

**Results:**

These methods produced methodological and technically comparable counts using KP, *rpoB*, and *adhE*. In mice fecal samples, we observed activity reductions ranging from 1.13 to 0.64 log_10_ DNA copies/mL. Interestingly, ciprofloxacin inhibited the recovery of VBNC-state cells while maintaining resuscitation and ethanol production after the removal of antibiotics in HiAlc *Kpn*, and we quantified the number of viable cells directly by PMA-ddPCR. This is a method for direct quantification that does not require an external standard curve reference.

**Discussion:**

This is a method for direct quantification that does not require an external standard curve reference. This method could be an innovative tool for quantifying viable cells of *K. pneumoniae*, with potential applications in quantifying bacterial cells in the VBNC state, resuscitation, and assessing the persistence of pathogens in clinical samples.

## Introduction

*Klebsiella pneumoniae* is a multi-drug-resistant pathogen in hospitals, and has become resistant to many widely used drugs, such as colistin, fosfomycin, and tigacycline (Bassetti et al., 2018; Alvear-Daza et al., 2021). Our previous research has shown that high alcohol-producing *K. pneumoniae* (HiAlc *Kpn*) in the gut microbiota could cause non-alcoholic fatty liver disease (NAFLD) and can enter a dormant state, which could be defined as viable but non-culturable (VBNC) state (Yuan et al., 2019; Alvear-Daza et al., 2021). This state is metabolically active but cannot be grown on conventional media (Pinto et al., 2015a). When exposed to adverse environments such as starvation, low temperature, irradiation, and antibiotic pressure, many bacteria can enter the VBNC state (Oliver, 2005; Wu et al., 2016; Zhang et al., 2021). Although bacteria in the VBNC state evade detection on standard media, they remain viable and can be revived by removing stress (Pinto et al., 2011). VBNC bacteria not only have higher antimicrobial and antibiotic resistance, but have even been found to produce virulence (Pinto et al., 2015b). Ciprofloxacin is a third-generation quinolone antibacterial drug with broad-spectrum antibacterial activity and excellent bactericidal effect. Previous studies have shown the inhibitory effects of imipenem, ciprofloxacin, polymyxin and bacteriophages on the recovery of VBNC state cells. It was found that ciprofloxacin had a significant inhibitory effect, and it was selected as the detection and counting agent for inhibiting the recovery of viable cells (Zhao et al., 2024).

Direct plate counting offers a straightforward way to differentiate between live and dead cells, whereas techniques that rely on molecular amplification are notably challenging, particularly when analyzing aquatic samples with low cell concentrations (Davis, 2014). Integrating propidium iodide staining with cell integrity assessments using an electronically programmed sorter has been suggested (Caron et al., 1998). A Previous study combined live/dead staining with microscopy or flow cytometry to distinguish viable from dead cells (Gonzalez-Escalona et al., 2006). However, the reliability of these methods often hinges on the operator’s experience, restricting their broad application (Strauber and Muller, 2010).

Quantitative real-time PCR (qPCR), when combined with treatments like propidium monoazide (PMA) or ethidium monoazide (EMA), is a possible alternative method for detecting viable cells (Nocker et al., 2006; Elizaquivel et al., 2014). Both PMA and EMA can penetrate cells with damaged membranes or attach to free DNA, which prevents the amplification of non-viable or damaged cells. This approach presumes that cells with intact membranes are viable. Because EMA treatment displays heightened cytotoxicity in viable cells compared with PMA, which leads to false-positive counts, PMA-qPCR has become a common method for distinguishing between viable and dead cells (Banihashemi et al., 2012; Wu et al., 2015; Zhao et al., 2022). This is important according to the guidelines for reference gene selection in qPCR experiments (Bustin et al., 2009). However, typically qPCR analyses are performed using one single copy gene. In addition, PCR efficiency and analysis parameters are often omitted in published literature. The average data of three single-copy genes are recommended for qPCR analysis.

Droplet digital PCR (ddPCR) is a microfluidic- or microdroplet-based method for clinical detection and quantification of microorganisms (Sun and Joyce, 2017; Li et al., 2018). This technique can precisely quantify the gene copy number without external reference such as a standard curve. During its operation, the ddPCR separates DNA samples into minuscule reaction droplets and amplified by PCR. Each droplet’s fluorescence is subsequently analyzed, which facilitates DNA quantification through the Poisson binomial distribution. Notably, ddPCR quantification of stably expressed single-copy genes yields robust data, even for samples with diluted concentrations. The data from ddPCR has shown strong correlation with qPCR findings, with PMA-ddPCR effectively quantifying bacterial survival in fecal samples (Gobert et al., 2018; Zhao et al., 2021; Feng et al., 2023).

In this work, we developed PMA-ddPCR to quantify VBNC cells using KP (encoding hemolysin protein), *rpoB* (encoding DNA-directed RNA polymerase subunit beta), and *adhE* (encoding bifunctional acetaldehyde-CoA/alcohol dehydrogenase), which are all single-copy genes in HiAlc *Kpn* (Li et al., 2021; Patole et al., 2021; Qiu et al., 2022; Feng et al., 2023). The results of ddPCR and qPCR showed that ddPCR combined with PMA incubation was a practical and effective method to count the HiAlc *Kpn* VBNC state cells gene. Furthermore, transmission electron microscopy (TEM) and fluorescence microscopy were used to visualize the appearance of the bacteria (Fittipaldi et al., 2012; Gou et al., 2024; Taylor et al., 2014), which revealed the morphological changes and viable cells in HiAlc *Kpn* VBNC state. In addition, ciprofloxacin inhibited the recovery of VBNC-state cells, and PMA-ddPCR allowed absolute quantification of viable bacteria while maintaining resuscitation and ethanol production after antibiotic removal. Our results show that combining ddPCR of three genes with PMA treatment offers a robust and efficient strategy for enumerating viable cells.

## Materials and methods

### Bacterial cultures

The ethanol production capacity of high alcohol-producing *Klebsiella pneumoniae* (HiAlc *Kpn*) W14 isolated from NAFLD patients was > 40mmol/L. After overnight culture, HiAlc *Kpn* was resuspended in Luria–Bertani (LB) broth and incubated at 37°C to 600 nm optical density (OD600) = 1.0. Finally, the culture was diluted in Artificial seawater (ASW) to a final concentration of 1×10^8^ CFU/mL. ASW was prepared by dissolving sea salt (40 g/L; Sigma-Aldrich, MO, USA) in water and then sterilizing it through a 0.22-μm membrane filter. LB agar plates were used to count culturable cells.

The cells were stored in ASW at 4°C for preparation of the VBNC state. Culturable cells were counted in ASW every 5 days in a 10 μL suspension on LB plates. No colony formation after 48 hours of incubation at 37°C is considered to have entered the VBNC state (Wu et al., 2015). In addition, the morphology of HiAlc *Kpn* and its VBNC state was observed using transmission electron microscopy (TEM) operated at 120 kV.

To test the sensitivity of detection, an ex-vivo experiment was conducted. C57BL/6J SPF mice fecal *in vitro* homogenization was added to serially diluted VBNC-state samples to identify the copy numbers. To detect ethanol production after resuscitation, alcohol concentration was measured three times using ethanol assay kit (BioVision, Milpitas, CA, USA). Then, the inhibitory effect of ciprofloxacin on resuscitation was examined. The solution with W14 VBNC state day 50 cells was resuspended in fresh YPD, and 3, 9, or 18 µg/mL ciprofloxacin was added, and the solution was shaken at 200 rpm at 37°C. Optical density was measured at 600 nm (OD600). After culturing for 6 hours, and the solution was washed once with PBS, new YPD was added, and the solution was then shaken at 200 rpm at 37°C overnight.

### Optimal conditions for PMA treatment

The PMA (Biotium, Hayward, CA, USA) application of *rpoB* single-copy gene were enumerated viable cells through qPCR or ddPCR methods, in order to inhibit the expansion of dead cells. The final concentrations of PMA were 5, 20, 50, 100 and 200-μM in ultrapure water. To optimize the PMA incubation time at lab temperature, a 650W double-ended halogen light source was used at 20cm distance from the sample tube for 15 minutes after 5, 10, 20 or 30 minutes of incubation without light. The sample was placed on ice during the lighting process to avoid excessive heating.

### DNA isolation

Genomic DNA was isolated from 200-μL cell suspensions using the Wizard Genomic DNA Purification Kit (Promega, Madison, WI, USA). Subsequently, 200-μL DNA preservation solution was added for overnight storage at 4°C.

### qPCR analysis and standard curve

The qPCR amplification mixture contained 1 μL template DNA, Premix Ex Taq (TaKaRa, Dalian, China), 0.25 μM forward and reverse primer for HiAlc *Kpn* W14 *KP*, *rpoB* or *adhE* single-copy genes (**Table 1**), and ultrapure water to 20 μL final volume. qPCR was performed on a QuantStudio 7 Flex (Applied Biosystems) instrument and the cycle as follows: initial PCR activation at 95°C for 30 s, followed by 40 cycles of denaturation at 94°C for 10 s and annealing/extension at 60°C for 45 s. If the Cq value is greater than 39, the enumeration results were considered negative. To prepare the standard curve, DNA was extracted from purified HiAlc *Kpn* W14 and serially diluted to make the standard curve. Using the initial sample concentration, the initial target DNA copy numbers in the DNA sample could be determined by calculating from the standard curve and using quantitative cycle (Cq).

**Table 1:** Oligonucleotides used in this study.

Primer	Sequence (5’-3’)
KP-F	CGATGCTACTTATCCCGACA
KP-R	AGCCGGTTGAGACGTAAAC
KP-probe	6FAM-CCGATTGAAAAACGCTCCGGGC-BHQ1
*rpoB*-F	CGAAATCGAAGGTTCCGGTAT
*rpoB*-R	ATCGTCCACTTCGCCTTTAC
*rpoB*-probe	6FAM-CCTGAGCAAAGACGACATCATCGAAGT-BHQ1
*adhE*-F	CTGTCAGAAGACGACACCTTT
*adhE*-R	GCTGTTGAAGTCGGGTTAGT
*adhE*-probe	6FAM-CCTATCGGCATCATCTGCGGTATCGTA-BHQ1

### ddPCR analysis

ddPCR was performed utilizing a TARGETING ONE droplet digital PCR system (TARGETING ONE Biotech Co., Ltd., Beijing, China), comprising a droplet generator and a chip reader. The PCR reaction was carried out in a total volume of 30 µL, comprising 15 µL reaction buffer, 1 µL DNA, 1.5 µL of each primer, and 11 µL ddH_2_O. The mixture was added to a disposable microfluidic chip. Then, 180 µL droplet generation oil was deposited onto the chip. Thereafter, the chip was loaded into a droplet manufacturing machine (TARGETING ONE Biotech Co.) to generate the droplets. The droplets were deposited into an eight-well PCR tube, and PCR amplification was facilitated within a Veriti 96-well thermal cycler (Applied Biosystems, CA, USA) under the following conditions: 95°C for 10 min; 40 cycles of 30 s at 94°C and annealing at 60°C for 1 min. The fluorescence signal was then detected with a Chip Reader (TARGETING ONE Biotech Co.) and analyzed with the Chip Reader R1 software.

### Live/dead staining

The examination of VBNC viability was conducted by performing live/dead staining as previously described (Zhao et al., 2021). The staining of the cells was accomplished by introducing a 1.5-µL mixture of SYTO9 and propidium iodide (1:1 v: v; Molecular Probes, Eugene, OR, USA) per 500 mL of suspension. Following an incubation period of 15 minutes in conditions of darkness, the stained cells were mounted on a glass slide. Subsequently, the cells were examined with fluorescence microscopy (KEYENCE BZ-X800).

### VBNC state strain resurrection

W14 VBNC state cells (at day 50) solution was re-suspended in fresh YPD (1:100, v/v), and 3,9 and 18 µg/mL ciprofloxacin was added at 200 rpm and shaken at 37°C. Measure the optical density of 600 nm (OD600). When incubation for 6 hours, the other group washed the imipenem antibiotic, washed it with PBS once time, and then added the new YPD, and shook it at 200 rpm and 37°C overnight.

### Statistical analysis

Figures were generated using GraphPad Prism software from three replicate values. Each replicate value (black dots) was derived as the mean value from a triplet measurement. The means and standard deviations (SDs) of the groups were analyzed using Student’s t-test. The differences among multiple means were analyzed through one-way or two-way analysis of variance (ANOVA). For each of the experiments in the study, the difference was deemed to be statistically significant if the p value was less than 0.05.

## Results

### Quantitative analysis of PMA concentration and incubation time by qPCR

**Figure 1:**
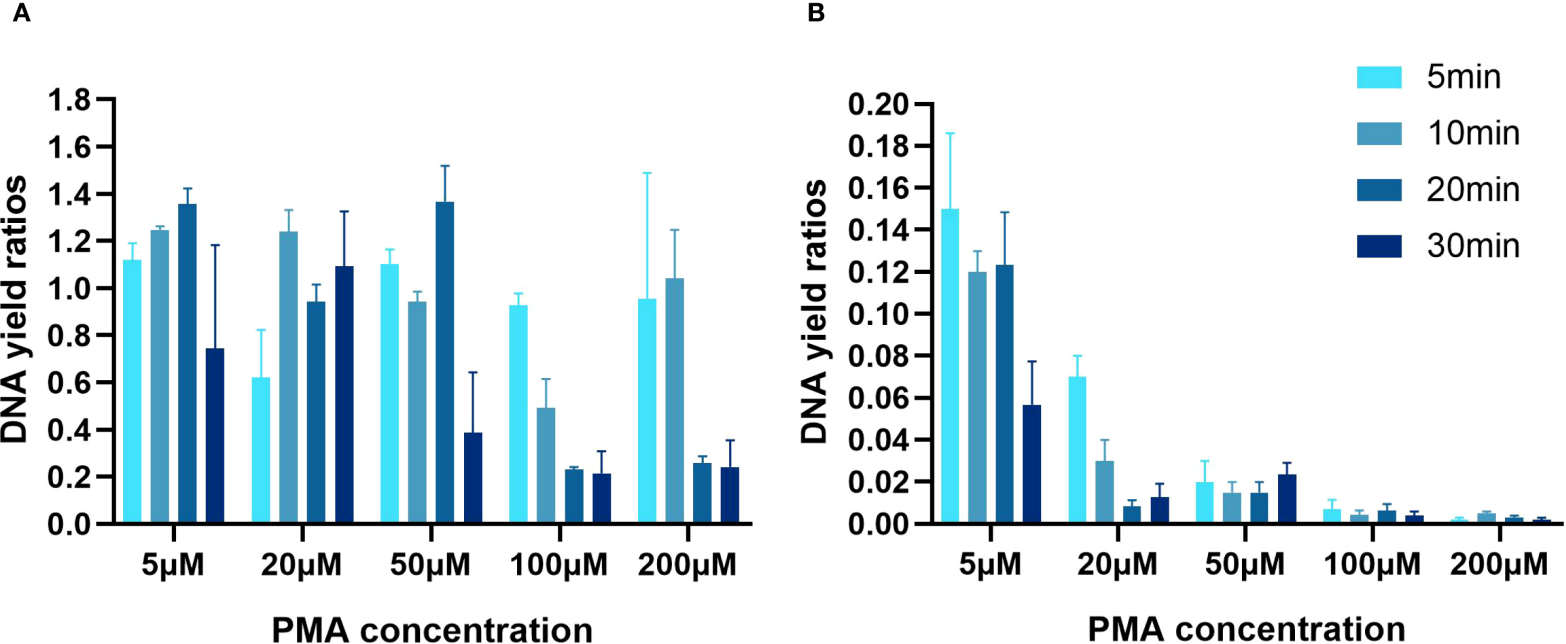
Determination of the optimal PMA concentration and incubation time for qPCR quantification. **(A)** Quantification of fresh cell suspensions. **(B)** Quantification of heat-killed cell suspensions. The scales in A and B were varied by a factor of 10.

The effects of varying PMA concentrations and incubation duration on the counting of viable cells differ between heat-killed and fresh cells (**Figure 1**). For fresh cells, when compared to the group without PMA treatment and with concentrations set at 5μM, 50μM, 100μM, and 200μM, and subjected to incubation in the dark for 5 and 10 minutes, the results indicate a low efficiency of PMA treatment in eliminating dead cells (**Figure 1A**). The concentration was 20μM, the proportion of viable bacteria fluctuated between 88% and 98% when incubated in the dark for 20 minutes. However, as PMA concentrations increased and incubation times lengthened, the count of viable bacterial DNA copies decreased.

For heat-killed cells (**Figure 1B**), the efficacy of PMA in suppressing DNA signals from dead cells increased with higher PMA concentrations. At 5μM of PMA with 5 minutes of incubation, there was an 85% suppression of the initial total cell DNA copies. When the PMA concentration exceeded 50μM, the signal detection rate gradually decreased. At a concentration of 20μM with 20 minutes of incubation, PMA inhibited 99.2% of signals from dead cells. Surprisingly, further increased in concentration and incubation duration did not affect the suppression efficiency for dead cells. Based on these observations, the optimal conditions for distinguishing between viable and dead bacterial cells were an incubation with 20µM PMA for 20 minutes in the absence of light.

### Sensitivity of qPCR and ddPCR quantification

The first step was to determine the specificity of the qPCR and ddPCR assays for HiAlc *Kpn* genes. The mean concentration of the HiAlc *Kpn* strain W14 cells was around 2.5×10^8^ CFU/mL, as measured by MacConkey agar plate counting. Chromosomal DNA extracted from W14 was used as the template for amplification and was prepared by 10-fold serial dilution. W14 DNA was used to construct standard curves for the quantification of the target genes KP, *rpoB*, and *adhE* by qPCR. The slopes were −4.50, −4.45, and −4.48 for KP, *rpoB*, and *adhE*, respectively. The lower limits of detection of total DNA based on mean Cq values were 37.73 ± 0.21, 38.04 ± 0.79, and 38.23 ± 0.56 for KP, *rpoB*, and *adhE*, respectively (**Table 2**).

**Table 2:** Quantification of KP, *rpoB*, and *adhE* genes in HiAlc *Kpn* W14 genomic DNA via qPCR and ddPCR with 10-fold serial dilutions.

			qPCR			
Dilutions	KP		*rpoB*		*adhE*	
	Cq mean	SD	Cq mean	SD	Cq mean	SD
10^0^	15.85	0.15	16.52	0.26	16.56	0.18
10^-1^	19.64	0.31	20.49	0.41	20.82	0.36
10^-2^	25.32	0.21	26.11	0.24	26.35	0.32
10^-3^	30.01	0.58	30.98	0.45	31.16	0.15
10^-4^	34.55	0.32	34.92	0.31	35.44	0.16
10^-5^	37.73	0.21	38.04	0.79	38.23	0.56
10^-6^	UD	UD	UD	UD	UD	UD
R^2^	0.995		0.992		0.991	
			ddPCR			
Dilutions	KP		*rpoB*		*adhE*	
	copies/μL	SD	copies/μL	SD	copies/μL	S
10^0^	ULOD	ULOD	ULOD	ULOD	ULOD	ULOD
10^-1^	52095.1	2145.89	31741.3	1200.87	12562.7	2598.23
10^-2^	4166.8	1021.32	3822.9	113.77	2954.4	1196.40
10^-3^	471.0	119.19	310.1	11.02	136.0	116.97
10^-4^	64.4	3.86	56.5	4.34	37.2	60.31
10^-5^	31.3	4.31	12.1	2.42	6.7	1.78
10^-6^	4.2	0.87	4.4	1.15	1.3	0.59
R^2^	0.999		0.999		0.981	

UD, undetected. ULOD, DNA concentration at which the signal of the assay was saturated.

To determine the lower limit of ddPCR detection, three single-copy genes were tested three times by ddPCR. A good linear relationship was observed. When total DNA was diluted to 10^-5^, 31.3 ± 4.31, 12.1 ± 2.42, and 6.7 ± 1.78 copies/µL of KP, *rpoB*, and *adhE* were detected, respectively. In addition, the lowest detection sensitivities were 4.2 ± 0.87, 4.4 ± 1.15, and 1.3 ± 0.59 copies/µL, respectively.

Both PCR methods, with R^2^ values between 0.981 and 0.999 for the three genes, showed good linear behavior in the quantification range. The number of DNA copies/µL the KP, *rpoB*, and *adhE* genes in samples not treated with PMA was similar using both qPCR and ddPCR.

### Morphological alterations and cellular quantification in the VBNC state

**Figure 2:**
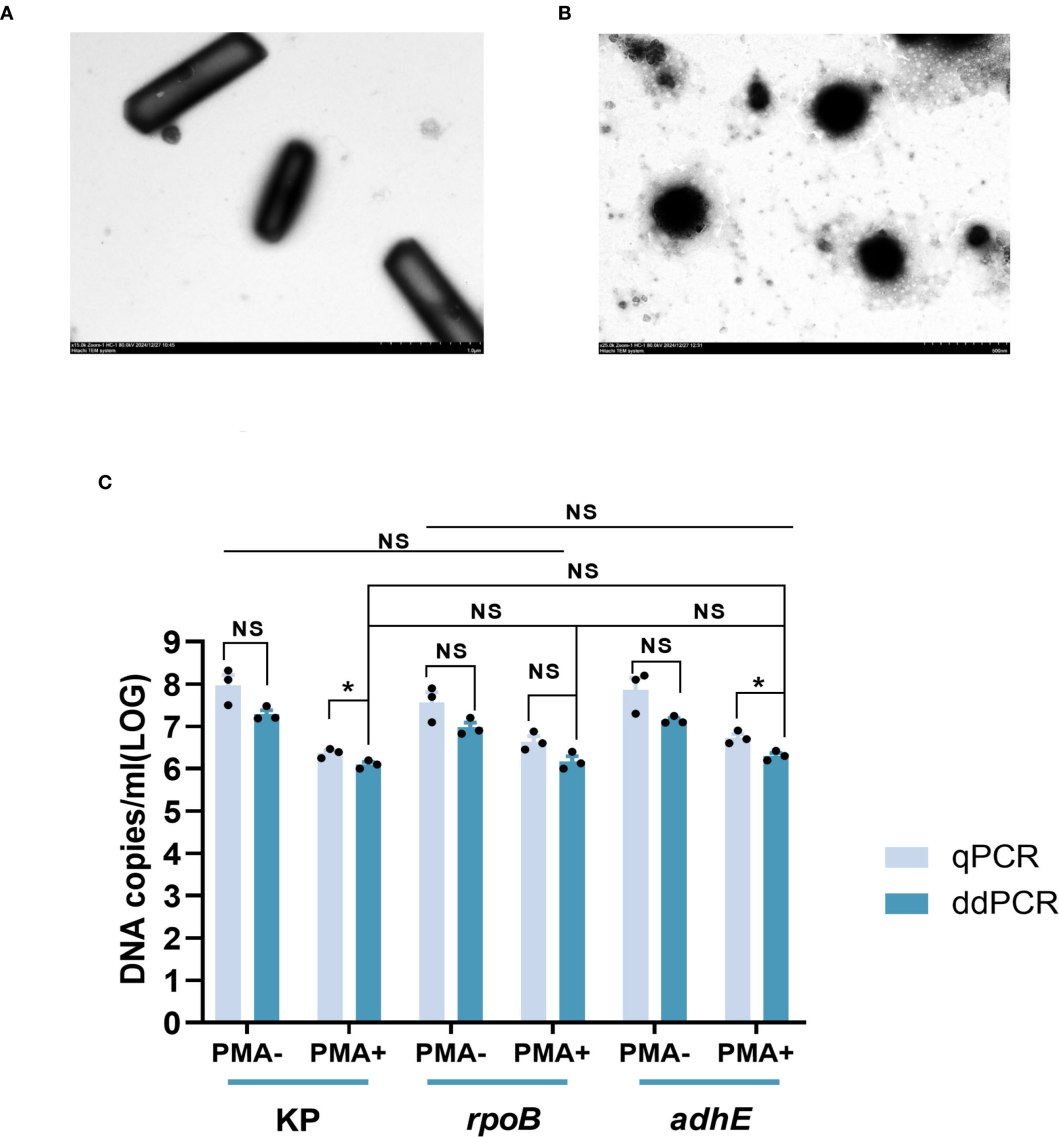
Electron microscopic observation of viable cells and VBNC state cells (at 50 days) and quantitative determination of total and viable cells by KP, *rpoB*, and *adhE* single-copy genes. **(A)** Viable cells Image obtained by electron microscopy. **(B)** VBNC state cells Image obtained by electron microscopy. **(C)** Quantification of cells in the VBNC state at 50 days. Total cells were measured without PMA treatment (PMA−) and viable cells were measured after PMA treatment (PMA+). qPCR, quantitative PCR; ddPCR, droplet digital PCR. Error bars represent the mean and standard error of measurement. NS, not significant, *p < 0.05, **p < 0.01, ***p < 0.001.

HiAlc *Kpn* strain W14 samples were taken on the 50^th^ day of VBNC state development. On the 50^th^ day under low-temperature and starvation, the cells entered the VBNC state. The cells underwent morphological transformations from bacillus to coccus and size reduction (**Figures 2A, B**). DNA copy quantification of VBNC cells was performed by qPCR and ddPCR. Three genes, KP, *rpoB* and *adhE*, were used as counting targets showed no significant difference. PCR was used to enumerate the total number of cells in the VBNC state and viable cells without or with PMA treatment. (**Figure 2C**). We reported the enumeration of DNA copies of three genes per mL based on one copy of each gene per cell.

Similar numbers of total cells (i.e., live + dead) were determined by ddPCR using KP and adhE (7.92 ± 0.16 and 7.05 ± 0.09 log_10_ DNA copies/mL, respectively), and slightly lower values were determined using rpoB (6.98 ± 0.20 log_10_ DNA copies/mL). Quantification of viable cells by ddPCR revealed 6.10 ± 0.18 and 6.18 ± 0.20 log_10_ DNA copies/mL using KP and rpoB, respectively, and a slightly higher value was determined using adhE (6.31 ± 0.11 log_10_ DNA copies/mL).

The loss of cell viability was assessed on the basis of the number of cells determined by PMA-qPCR or PMA-ddPCR. Using qPCR, the viability losses determined using KP, rpoB, and adhE were 1.60, 0.92, and 1.13 log_10_ DNA copies/mL, respectively. Using ddPCR, the viability losses determined using KP, rpoB, and adhE were 1.19, 0.80, and 0.84 log_10_ DNA copies/mL, respectively.

### Quantification of viable cells during VBNC state development

**Figure 3:**
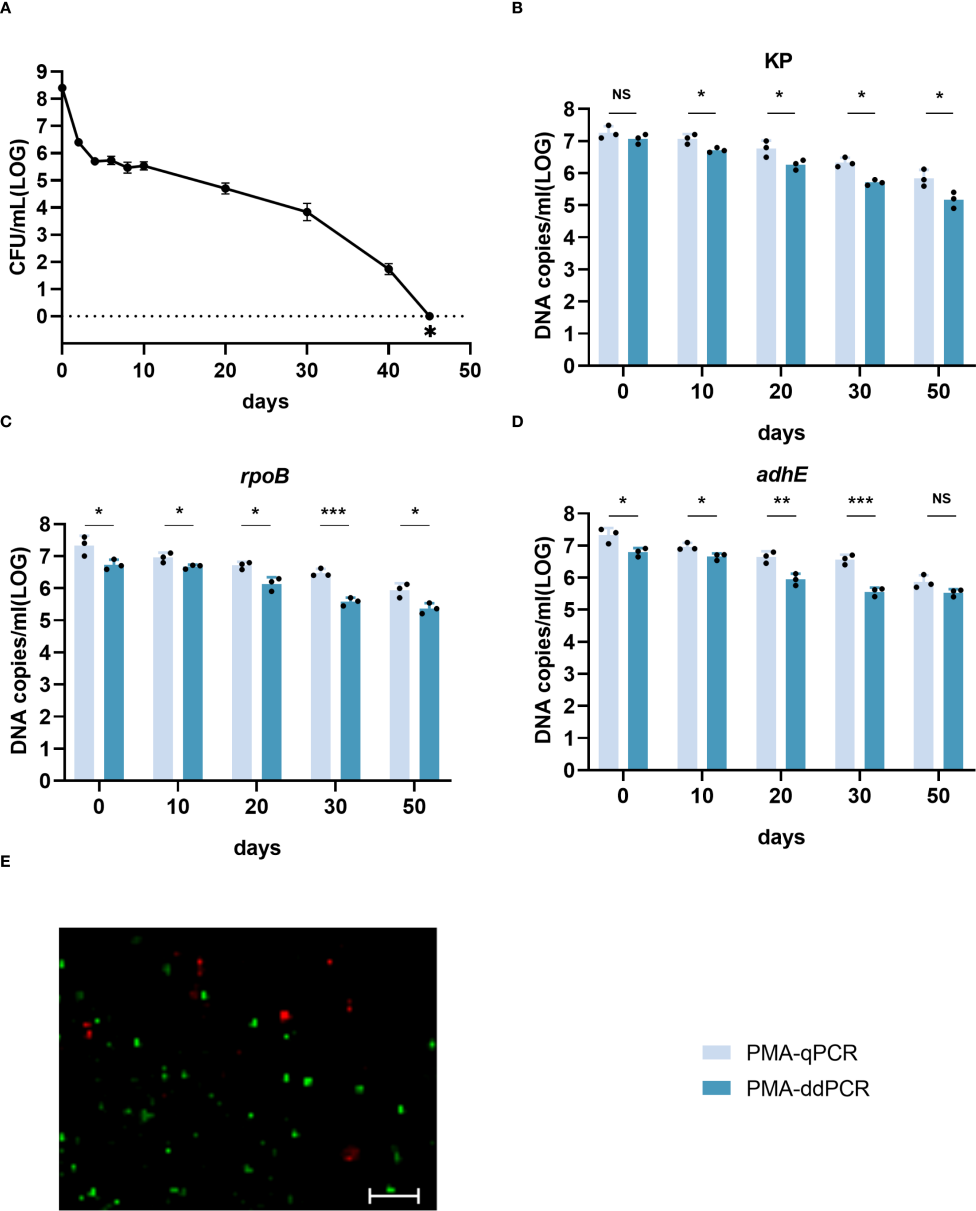
Enumeration of W14 viable cells by qPCR and ddPCR during the development of the VBNC state. **(A)** W14 Culturable cell counts throughout the progression of VBNC state. ∗ indicates that the culturable cell count was less than 1 colony-forming unit/mL. **(B–D)** DNA copies/mL quantified using the genes KP, *rpoB*, and *adhE* after PMA treatment. NS, not significant, *p < 0.05, **p < 0.01, ***p < 0.001. **(E)** Live/dead staining of cells of HiAlc *Kpn* W14 on day 50. Viable cells, which are only stained by SYTO9, appear green; dead bacteria appear red.

The development of the HiAlc *Kpn* W14 VBNC state involves a systematic process. PMA-treated and untreated cultures were collected on days 0, 10, 20, 30 and 50. qPCR and ddPCR were used to extract and determine cellular DNA. Culturable cell counts gradually decreased, reaching zero after 45 days. (**Figure 3A**). Viable cell counts were confirmed by live/dead staining, PMA-qPCR, and PMA-ddPCR (**Figure 3**). The viable cell counts determined by PMA-qPCR and PMA-ddPCR using KP, rpoB, and adhE showed a slow decrease from day 0 to day 50, and ranged from 6.59–7.60 log_10_ DNA copies/mL to 4.90–6.12 log_10_ DNA copies/mL (**Figures 3B–D**). PMA-ddPCR yielded a marginally lower number of DNA copies/mL than PMA-qPCR, and the qPCR values had a higher variance than the ddPCR data. Thus, ddPCR was more reproducible than qPCR for counting gene copies.

### Quantification of VBNC cells in mice fecal samples

**Figure 4:**
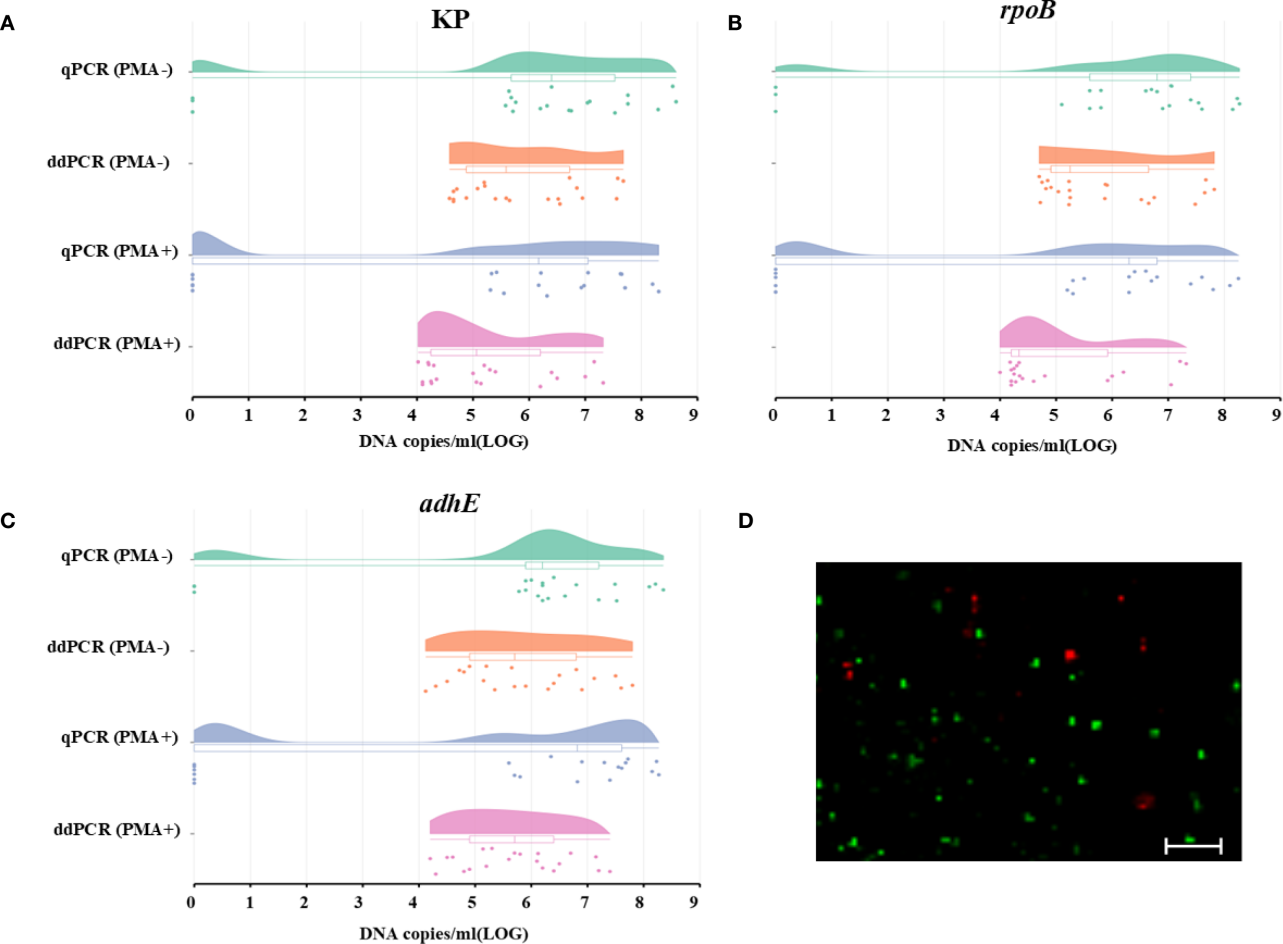
Quantification of total and viable cells using the genes KP, *rpoB*, and *adhE* to analyze the HiAlc *Kpn* W14 VBNC state in mice fecal samples. **(A–C)** DNA copies/mL were measured using the genes KP, *rpoB*, and *adhE* to analyze total and viable cells. Total cell counts were obtained without PMA treatment, and viable cell counts were obtained after PMA treatment. The dots represent the DNA copies/ml (LOG) of the three single-copy genes in the fecal samples, while the bars represent the dispersion of each data. **(D)** Live/dead staining of cells in the HiAlc *Kpn* W14 VBNC state in mice fecal samples (scale bar = 20 mm). Viable cells, which are only stained with SYTO9, appear green; dead bacteria appear red.

The applicability and sensitivity of qPCR and ddPCR for VBNC state cells were tested using mice fecal samples. The VBNC-state cells on the 50^th^ day were diluted, and each diluent was added to homogenized mice fecal samples. When total HiAlc *Kpn* cells were measured in samples not treated with PMA, neither qPCR nor ddPCR data showed significant differences, which confirmed good consistency between the two methods (**Figure 4**).

The quantitative results for the total cell count (both live and dead) in PMA-untreated samples by qPCR were 5.88 ± 2.64, 5.87 ± 2.61, and 5.77 ± 2.54 log_10_ DNA copies/mL for KP, rpoB, and adhE, respectively. The total cell counts quantified by ddPCR were comparable across different targets, yielding 5.86 ± 1.08 log_10_DNA copies/mL for KP, 5.85 ± 1.09 log_10_ DNA copies/mL for *rpoB*, and 5.87 ± 1.14 log_10_ DNA copies/mL for *adhE*.

In the PMA-treated samples, 15 out of 21 samples were successfully quantified using qPCR. Consistent with expectations, the viable bacterial count was marginally lower than the total count and was nearly at the lower detection limit of qPCR. However, all samples were analyzed by ddPCR. The precision was greatly improved and the lower limit of detection was decreased. The mean values of qPCR and ddPCR were 5.01, 4.85, and 5.40 log_10_ DNA copies/mL for KP, rpoB, and adhE, respectively. The lower limits of ddPCR (1.29, 1.20, and 1.40 log_10_ DNA copies/mL for KP, rpoB, and adhE, respectively) were higher than those of qPCR, which supported the results obtained from PMA-untreated samples.

The total number of cells in mice fecal samples was quantified using qPCR or ddPCR without PMA treatment, while the number of live VBNC cells was determined by qPCR or ddPCR combined with PMA treatment. The viability loss of VBNC cells was assessed by comparing the results obtained from PMA-qPCR or PMA-ddPCR with those from qPCR or ddPCR. For qPCR and ddPCR, the mean values for the loss of VBNC viability were 0.86, 1.01, and 0.42 log_10_ DNA copies/mL KP, rpoB, and adhE, respectively.

### Quantification of viable W14 cells in resuscitation

**Figure 5:**
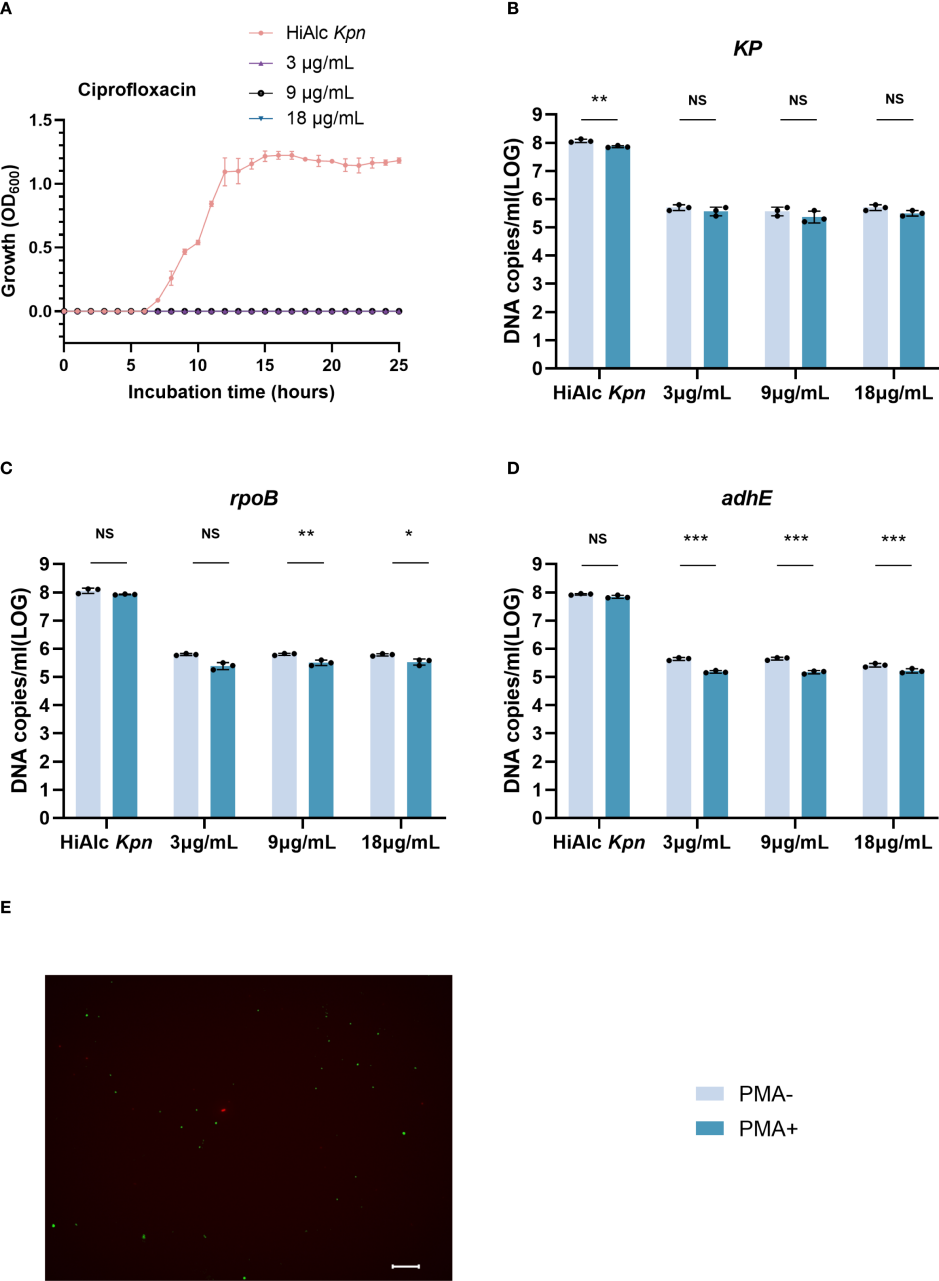
Ciprofloxacin (3, 9, and 18 μg/mL) was quantified by ddPCR to maintain resuscitation of viable cells in VBNC state. **(A)** VBNC state resuscitation OD600 growth curve for HiAlc *Kpn* and under addition of 3, 9, and 18 μg/mL ciprofloxacin. **(B–D)** DNA copies/mL were measured using the genes KP, *rpoB*, and *adhE* to analyze viable and total cells after 6 hours of resuscitation. NS, not significant, *p < 0.05, **p < 0.01, ***p < 0.001. **(E)** Live/dead staining of cells in the VBNC state after 6 hours of resuscitation with ciprofloxacin (scale bar = 20 mm). Viable cells, which are only stained with SYTO9, appear green; dead bacteria appear red.

To evaluate the quantification of HiAlc *Kpn* W14 viable VBNC cells (at 50 days) during resuscitation, we added 3, 9, or 18 µg/mL of ciprofloxacin and quantified viable cells at 6 hours of resuscitation (**Figure 5**). It was found that the W14 strain began to recover at 6 hours, and the addition of ciprofloxacin could significantly inhibited VBNC-state cell resuscitation. Total cells and viable cells significantly decreased, and recovery effect did not significantly differ among concentrations. The total number of cells added with different concentrations of ciprofloxacin were 5.66 ± 0.12, 5.79 ± 0.13, and 5.57 ± 0.12 log_10_ DNA copies/mL for KP, rpoB, and adhE, respectively. For these three genes, PMA-ddPCR quantified 5.48 ± 0.16, 5.47 ± 0.12 and 5.19 ± 0.06 log_10_ DNA copies/mL, respectively, of viable cells. Live and dead staining also demonstrated the presence of viable cells when exposed to ciprofloxacin for 6 hours. After washing imipenem 6 hours after resuscitation, this strain could still be resuscitated, and the ethanol concentration was still 44.19 ± 2.3 mmol/L after resuscitation.

## Discussion

Quantifying viable versus dead bacterial cells in the VBNC state necessitates molecular-level identification of pathogenic bacteria. Such methods can also elucidate the correlation between ciprofloxacin resistance, alcohol production, and HiAlc *Kpn* survival during VBNC-state cell resuscitation. Therefore, specific quantitative tools to distinguish and quantify viable cells from dead cells are required. Methods based on cell activity and nucleic acid synthesis can serve as qualitative detectors of viable cells (Zhao et al., 2021). Every viable cell has chromosome DNA that can be quantified by enumerating the individual copy genes within the DNA. In this study, we optimized PMA concentration and culture duration, then evaluated the efficacy of PMA-ddPCR for quantitation of HiAlc *Kpn* W14 viable cells in VBNC state cells and resuscitation.

The conventional PCR techniques usually only indicate the presence or absence of gene amplification, but cannot provide direct evidence of cell viability. In such cases, PMA treatment needs to be combined to detect living cells. The PMA treatment can bind to damaged membrane DNA and inhibit its amplification (Nocker et al., 2006) (Arvaniti et al., 2025). Through qPCR and ddPCR amplification results, single-copy genes of viable cells can be detected. This study confirmed that the amplification efficiency of qPCR was combined with the PMA concentration and the incubation time in HiAlc *Kpn* W14. The PMA concentration was detected when cells are alive. When PMA was used to detect viable cells and the concentration exceeds 50 µM, a higher concentration of PMA will reduce the detectable viable HiAlc *Kpn* W14 DNA. At a PMA concentration of 20 µM, no marked difference was noted in living cells after 20 minutes. However, a distinct time and concentration-dependent relationship was evident in dead cells, with up to 99% of DNA remaining unamplified. This result is consistent with the studies of *Vibrio cholerae, Campylobacter, and Mycobacterium* (Fittipaldi et al., 2012; Kruger et al., 2014; Seinige et al., 2014; Wu et al., 2016). This detection method can be applied to the detection of living cells in the VBNC state, which can improve the sensitivity of pathogen detection in food, clinical and environmental contamination monitoring, and outbreak warning.

The minimum number of positive gene copies is crucial for qPCR and ddPCR differences. ddPCR detected a lower number of gene copies than qPCR. For mice fecal VBNC samples, ddPCR detection limit was 4.02 to 4.7 log_10_ DNA copies/mL, higher than qPCR. The significance of this difference lies in the fact that ddPCR can provide accurate results even with a low number of VBNC cells. This allowed VBNC cells to be quantified by ddPCR in more low concentration samples. It has been previously reported that qPCR cannot accurately quantify low numbers of VBNC cells (Lahteinen et al., 2014). Also consistent with previously published literature, ddPCR is better than qPCR at quantifying low levels of target molecules (Gobert et al., 2018; Xu et al., 2021).

To assess the number of cells in the VBNC state in this study, we selected three single-copy genes. These genes will be used in parallel to validate quantitative PCR methods for gene copy numbering and to compare outcomes when different genes are tested with different primers and probes. Simultaneous qPCR and ddPCR used target genes (KP, *rpoB*, and *adhE*) to successfully quantify DNA copy numbers in both total and viable cells, with no significant differences between genes. The small differences in the mean Cq values of the three genes probably resulted from different qPCR efficiency among primers, but the Cq values were similar, which verified the feasibility of this method. However, at each time point in VBNC state development, the DNA copy number confidence interval measured by qPCR was wider than that of ddPCR; this may be due to the fact that ddPCR is an absolute counting method, whereas qPCR is based on amplification efficiency, Cq values, and reference-based calculations. We obtained similar gene copy counts when KP, *rpoB*, and *adhE* were used, suggesting that these genes and the primers tested in this study could be used to count cells in the VBNC state.

It is crucial to determine whether *Kpn* strains in the VBNC state could be killed or whether the resuscitation process could be inhibited to control their potential risk. When the cells were resuscitated in the VBNC state and treated with different concentrations of ciprofloxacin, the bacteria did not grow. The viable cell copy number could be detected by three single-copy genes using PMA-ddPCR. When ciprofloxacin was washed, the bacteria were still recovering, and cells found in the VBNC state were not killed but their recovery was inhibited. After recovery, the ethanol production of VBNC cells could still be restored in viable cells. This indicated that HiAlc *Kpn* was resistant to ciprofloxacin in the VBNC state and maintained its ethanol-producing properties. Therefore, the absolute quantification of viable cells plays an essential role in the detection of VBNC-state cells.

We established and evaluated a quantitative PCR-based approach to count viable but non-culturable HiAlc *Kpn* W14 by counting single-copy chromosome genes in VBNC-state cells. PMA effectively excluded the dead cells’ chromosomal DNA. Better counting results were obtained for VBNC-state cells with ddPCR combined with PMA compared with qPCR. This method could be used to analyze VBNC-state and low-concentration samples inhibited by antibiotics, and to detect and monitor VBNC-state cells in clinical samples.

## Conclusions

When tested with pure cultures of HiAlc *Kpn* W14 standard strains, ddPCR showed a lower limit of detection than qPCR, increasing the quantitative accuracy and sensitivity. In the complex matrix of mice fecal samples, ddPCR showed a significantly higher detection rate, indicating its potential to achieve stable detection of gene targets in complex matrices. The occurrence, development, and resuscitation of the VBNC state represent a complete bacterial evasion process. The HiAlc *Kpn* three single-copy genes were simultaneously applied in both pure cultures and mice fecal samples, and it was able to accurately count the number of viable cells during the antibiotic inhibition and recovery process. The results indicate that ddPCR has great potential in epidemiological monitoring. In addition, VBNC state *Kpn* cells retain ethanol producing characteristics after resuscitation. The presence of VBNC *Kpn* in polluted water environments and its resuscitation in the human gut to produce ethanol can contribute to the occurrence and development of NAFLD. Under the influence of antibiotic resistance and diseases related to the gut microbiota, the detection of VBNC pathogens has significant implications for early patient detection, hospital epidemic monitoring, and food/water safety testing, thus preparing for future disease outbreaks.

## Limitations of the study

This study has some limitations. The VBNC state cells morphology change and the number of viable cells were observed using Negative Staining for TEM and fluorescence microscopy. Subsequently, confocal laser scanning microscopy will be applied to observe the biofilm structure in the VBNC state. In addition, through the RNA-seq and metabolome analysis of the VBNC state, regulatory factors and regulatory pathways related to starvation and low temperature were observed (data not shown). More supportive experiments such as biochemical analysis, protein profiling or fatty acid analysis are needed to validate the research results and provide stronger evidence.

## Data Availability

The original contributions presented in the study are included in the article/supplementary material. Further inquiries can be directed to the corresponding authors.
